# Association between Occupational Characteristics and Overweight and Obesity among Working Korean Women: The 2010–2015 Korea National Health and Nutrition Examination Survey

**DOI:** 10.3390/ijerph17051585

**Published:** 2020-02-29

**Authors:** Mi-Jung Eum, Hye-Sun Jung

**Affiliations:** 1 Department of Public Health, Graduate School, The Catholic University of Korea, 222 Banpo-daero, Seocho-gu, Seoul 06591, Korea; 2 Department of Preventive Medicine, College of Medicine, The Catholic University of Korea, 222 Banpo-daero, Seocho-gu, Seoul 06591, Korea

**Keywords:** overweight, obesity, occupational characteristics, working hours, shift work, night work, female workers

## Abstract

Associations between several occupational characteristics and obesity are not fully elucidated in Korean working populations, especially in females. This study investigated associations between occupational characteristics and overweight/obesity among Korean women. Data on 2090 female workers (the mean age was 38.8 ± 0.2 years), extracted from Korean National Health and Nutrition Examination Surveys in 2010–2015, were analyzed and showed that 6.8% of subjects were underweight, 50.8% had normal weight, 20.1% were overweight, and 22.2% were individuals with obesity. Multiple regression analysis was performed to examine associations between occupational characteristics and overweight/obesity, after controlling for demographic, behavioral, and health-related characteristics. The reference group was normal weight. Working hours were strongly associated with overweight/obesity. The odds ratio (OR) of obesity in women who worked for ≥60 h per week was 2.68 (95% confidence interval: [CI] 2.13–3.36) compared with those who worked for <40 h. Night/shift workers were 1.21 times (95% confidence interval: [CI] 1.01–1.45) more likely to experience obesity than day or evening workers. In conclusion, obesity rates increase among female workers with longer working hours and those who work at night or in shifts. Occupational characteristics should be considered in the prevention of obesity among working women.

## 1. Introduction

Globally, obesity is recognized as an important public health issue. At least 2.8 million deaths occur due to excessive weight or obesity annually [1] According to a report by the Organization for Economic Co-operation and Development (OECD), the prevalence of obesity among adults was 19.5% in 2015, indicating that one of four adults was an individual with obesity, and the prevalence of obesity is expected to increase at least until 2030 [2]. In Korea, adults showed that the prevalence of obesity (defined as a BMI ≥ 25 kg/m^2^) was 35.5% in 2016 [3]. It showed a consistently increasing trend among both men and women over the past 10 years. [4].

Obesity causes a variety of very serious health problems in adulthood and increases risks of hypertension, type 2 diabetes, cardiovascular disease, and mortality [5,6,7,8]. In addition, it is a factor that gradually increases direct and indirect healthcare costs and decreases productivity [9]. Andrea et al. performed a systematic review of 50 studies on indirect costs of overweight and obesity and found that indirect costs were increased among overweight workers, and the costs were even higher among workers with obesity due to decreased productivity [10]. In modern society, the prevalence of overweight and obesity is steadily increasing due to the automation in jobs and an increase in unhealthy behaviors, such as the lack of physical activity, smoking, drinking, and unbalanced nutritional intake. Therefore, obesity should be carefully examined, as well as other modifiable factors, such as blood pressure, blood glucose level, smoking, and drinking [6]. 

In the past several years, many countries have promoted a variety of strategies by establishing new regulations and policies aimed at eliminating obesity [2]. In July 2018, the Korean Ministry of Health and Welfare presented a “Comprehensive Plan for National Obesity Management,” which describes detailed strategies across four areas: nutrition, exercise, obesity treatment, and improved awareness. The plan shows the strong will of the government to prevent and manage obesity by taking aggressive measures [11]. 

Occupational characteristics are just a few of the diverse factors that influence obesity. Weight gain is reported to occur more frequently among shift workers than among day workers [12,13,14]. In a prospective cohort study conducted among Chinese workers, permanent irregular night work was more likely to be associated with overweight and abdominal obesity than shift work [15]. Additionally, a study that assessed data in Australia found gender differences in the patterns of overweight and obesity according to the job category [16]. A study conducted among workers in the United States (US) also reported that the prevalence of obesity varied according to the job category [17]. According to a study in the US using 2004–2011 data from the National Health Interview Survey, the annual obesity prevalence greatly increased among white-collar workers but changed little among blue-collar workers over the years [18]. A systematic review of 47 studies on the relationship between occupational factors and obesity revealed that there was a positive relationship between working for long hours and the outcome of overweight in approximately 70% of the studies [19]. According to Jang et al., the likelihood of obesity increased by 1.65 times among Korean male manual workers working ≥60 h per week, but not significantly among females [20]. As shown above, working for extended hours negatively affects health in various ways [21,22,23,24]. Korea, however, ranks second among the OECD countries in total working hours, with mean annual hours worked of 1993, which is 259 h higher than the OECD average of 1734 h [25]. Persistently working for extended hours not only impairs the worker’s health but also increases the risk of injury and occupational accidents [26,27]. 

The studies described above show the association between occupational characteristics and obesity among workers. However, the results were inconsistent between men and women. Regarding obesity among women in particular, sex hormone-related factors like menopause [28] are important to take into account, but very few studies have considered such variables.

Employees spend more than a third of the day at the workplace. Recently, although increasingly more women are engaged in economic activities, women are more likely than men to work both at the workplace and at home since women still take more responsibility for housework, pregnancy, child rearing, and so on. It is reported that in dual-earner couple households, compared to husbands, wives spend 7.4 times more time doing housework and 3.5 times more time performing child rearing duties during the week, and 4.3 times more time doing housework, 1.7 times more time performing child rearing activities, and 1.1 times less time engaging in leisure activities during weekends [29]. Lee et al. found that fatigue scores increased with total working hours among women [30]. Furthermore, weight gain was found to be associated with women working to earn money and also being responsible for housework [31]. Thus, various occupational characteristics including working hours are related with the health of working women and warrant the attention of society.

Accordingly, the present study aimed to investigate the association between occupational characteristics and overweight/obesity among working women by examining the variables, taking female characteristics into account.

## 2. Materials and Methods

### 2.1. Study Population

The present study analyzed the data integrated by combining raw data from the 2010–2015 Korean National Health and Nutrition Examination Surveys (KNHANES) [32]. The KNHANES is a nationwide cross-sectional survey conducted in Korea on health and nutrition since 1998. The most recent census data that were available at the time of sampling were used as the KNHANES sampling framework so that each sample would be representative of Korean citizens aged ≥1 year and living in Korea. Samples were obtained by means of 2-stage stratified sampling with the enumeration area as the primary sampling unit and household as the secondary sampling unit.

Health surveys and screening were performed at mobile screening centers, and nutritional surveys were performed during home visits. Health and nutrition surveys were completed either by interview or self-administered questionnaires. For a health screening, measurements, observation, and specimen analyses were performed.

**Figure 1:**
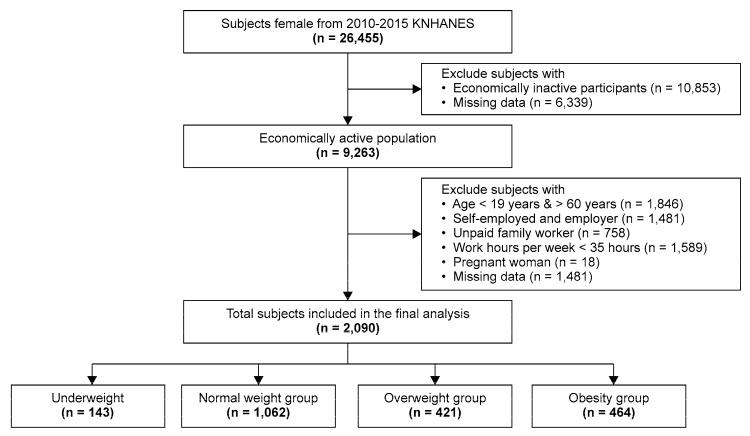
Flow chart of patient selection in the present study.

The initial sample contained 48,482 individuals. The focus of the present study was wage workers; thus, those not participating in economic activity (*n* = 15,501), self-employed or business owners (*n* = 1481), and those caring for a family without compensation (*n* = 758) were excluded from the study. In addition, individuals younger than 19 years or older than 60 years (*n* = 1846) and those working for less than 35 h per week were excluded from the study based on the assumption that they did not work 5 or more days per week. Individuals without a diagnosis of obesity or with incomplete survey data and currently pregnant women (*n* = 18) were also excluded. The final sample contained 2090 subjects (Figure 1).

### 2.2. Measurements and Variable Definitions

#### 2.2.1. Overweight and Obesity

The BMI, a universal index of overweight/obesity, was calculated by dividing the weight (kg) by the squared value of the height (m). In this study, the BMI was calculated using the weights and heights measured during health screening. The World Health Organization (WHO) classifies persons with a BMI of 25 kg/m^2^ or more but less than 30 kg/m^2^ as overweight and a BMI of ≥30 kg/m^2^ as individuals with obesity [33]. However, Asians with a BMI of ≥25 kg/m^2^ are classified as individuals with obesity according to the WHO Asia-Pacific perspective [34]. In this study, subjects with a BMI of <18.5 kg/m^2^ were classified as underweight, a BMI of ≥18.5 kg/m^2^ and <23 kg/m^2^ as normal weight, a BMI of ≥23 kg/m^2^ and <25 kg/m^2^ as overweight, and a BMI of ≥25 kg/m^2^ as individuals with obesity [34]. 

#### 2.2.2. Occupation-Related Variables 

Average weekly working hours were categorized into four groups (<40, 40–49, 50–59, and ≥60), based on the response to the item “On average, for how many hours per week do you work at your job, including overtime?”

The work pattern was classified in the following manner based on the response to the item “Primarily, do you work during the day or during other times in a day?”. Subjects whose responses were “Between 6 in the morning and 6 in the evening” were categorized as day workers, “Between 2 in the afternoon and midnight” as evening workers, “Between 9 at night and 8 the following morning” as night workers, and “Regular day-night shift” or “24-hour shift” as shift workers. Of the 4 categories, day and evening workers were combined into the “day or evening worker” category and night and shift workers into the “night or shift worker” category.

The working status (permanent, temporary, or daily) was determined based on the response to “Which one of the following three categories best describes your working status?”.

The job category was based on the occupation subjects provided in response to “What is your occupation?”. The responses were classified in accordance with the Korean Standard Classification of Occupations and then finally categorized into white-collar (including managers, professionals, and office workers), pink-collar (including service employees and salespersons), or blue-collar (including farming/forestry/fishery, skilled workers, equipment and machine operators, and assembly workers) jobs.

#### 2.2.3. Other Independent Variables 

For demographic characteristics, the following variables were used: age (19–29, 30–39, 40–49, and ≥50 years), educational level (some elementary school or finished elementary school, finished middle school, finished high school, and graduated from college or higher educational institutions), income level (1st, 2nd, 3rd, and 4th quartiles), and marital status (married and single). Behavioral characteristics, smoking (yes, no), drinking (yes, no), physical activity (yes, no), daily calorie intake (<2000, 2000–2499, and ≥2500 kcal), and daily sleep duration (<7, ≥7 h) were included. Health-related characteristics included hypertension (yes, no), diabetes (yes, no), dyslipidemia (yes, no), and menopause (yes, no).

### 2.3. Statistical Analysis 

Before data analysis, all data were processed by designing a complex sample by applying the weights presented in the guidelines of the Korea Centers for Disease Control (KCDC). The chi-squared test was performed to examine the weight category of overweight and obesity according to the subjects’ demographic, behavioral, health-related, and occupational characteristics. To determine differences among the groups for variables with significant differences in the chi-square test, a post hoc comparison analysis was performed by applying Bonferroni’s correction. To compensate for multiple tests, the *p*-value of each test was divided by the number of comparisons. A multiple logistic regression model was computed to estimate adjusted odds ratios (aORs) and their corresponding 95% confidence intervals (CIs) to investigate the relationships with overweight and obesity, with normal weight as the reference category. In the model, age, education level, income level, marital status, smoking, physical activity, daily calorie intake, daily sleep duration, hypertension, diabetes, dyslipidemia, and menopausal status were used as control variables. All statistical analyses were performed using SPSS version 23 (IBM Corp., Armonk, NY, USA). Analysis items with two-tailed *p*-values less than 0.05 were considered statistically significant.

### 2.4. Ethical Considerations

All versions of the KNHANES were executed by the KCDC, and all subjects provided written informed consent before participating in the survey. The data were de-identified before the analyses. The protocol of the present study was approved by the institutional review board of the Catholic University (MIRB-MYUN20200103-002; approval date: 3 January, 2020) and carried out following the rules of the Declaration of Helsinki.

## 3. Results

### 3.1. Weight Category and Demographic, Behavioral, Health-Related, and Occupational Characteristics 

Table 1 presents frequencies and other descriptive statistics of demographic, behavioral, health-related, and occupational characteristics of the subjects. Regarding weight, 6.8% of the subjects were underweight, 50.8% had a normal weight, 20.1% were overweight, and 22.2% were individuals with obesity. The mean age was 38.8 ± 0.2 years, the mean sleep duration was 6.8 ± 0.02 h per day, and the mean working duration was 45.4 ± 0.1 h per week.

Among subjects aged ≥50 years, the likelihood to be overweight or an individual with obesity was higher among those who had an education level of no more than elementary school, were married, slept less than seven hours per day, had diabetes and dyslipidemia, and were post-menopausal. Regarding occupational characteristics, the likelihood to be overweight or an individual with obesity was higher among subjects working for ≥60 h per week, and those working a blue-collar job.

### 3.2. Association between Weight Category and Demographic, Behavioral, Health-Related, and Occupational Characteristics

The associations between weight category and demographic, behavioral, health-related, and occupational characteristics are presented in Table 2. The weight category was significantly associated with age, education level, income level, marital status, smoking, physical activity, daily sleep duration, daily calorie intake, hypertension, diabetes, dyslipidemia, menopause, the number of working hours per week, work pattern, working status, and job category. Bonferroni’s correction was performed for multiple comparisons among the groups for variables showing significant differences. 

### 3.3. Multiple Logistic Regression Analysis of the Association between Occupational Characteristics and Overweight/Obesity

Table 3 shows the association between occupational characteristics and overweight/obesity. Multiple regression analysis was performed to assess the association between the two after controlling for demographic, behavioral, and health-related characteristics. The results showed that the weekly number of working hours, work pattern, and job category were significant factors for being overweight compared to normal weight, and that the weekly number of working hours and work pattern were significant factors for obesity. Specifically, the OR of obesity in women who worked for 40–49 h per week was 1.56 (95% CI, 1.17–2.08), the OR of obesity in women who worked for 50–59 h per week was 1.38 (95% CI, 1.03–1.85), and the OR of obesity in women who worked for ≥60 h per week was 2.68 (95% CI, 2.13–3.36), compared with those working for <40 h per week. In addition, night or shift workers were 1.21 times (95% CI, 1.01–1.45) more likely to have obesity than day or evening workers.

## 4. Discussion

Our study showed that two-fifths of working women in Korea were overweight or obese and that 22.2% were individuals with obesity. The analysis performed to assess the association between weight category and demographic, behavioral, health-related, and occupational characteristics showed the existence of a significant association in all variables except drinking and perceived stress level. The multiple logistic regression model on the association between occupational characteristics and overweight/obesity showed that of several occupational characteristics, the number of working hours per week increased the likelihood of overweight or obesity. The mean weekly number of working hours among all subjects was 45.4 and 8.8% worked for ≥60 h per week. Compared with subjects who worked for <40 h per week, those who worked for ≥40 h tended to be more likely to being obese. In particular, the OR of obesity among those working for ≥60 h per week was 2.68.

In a study conducted among adult wage workers in the US, the OR of obesity among those who worked for ≥50 h per week was 1.32, compared with those who worked for <30 h [35]. Working overtime is reported to be associated with weight gain among both middle-aged men and women [36]; however, some studies have found a significant difference among women only [37]. In a study conducted in Australia with a total of 9276 working women aged 45–50 years, the rate of weight gain increased as the number of working hours increased and the association between the number of working hours and weight gain was stronger in higher percentiles of the weight gain distribution [38]. A study conducted in Korea reported that the likelihood of overweight or obesity increased by 3.82 times among women aged >50 years and working for ≥9 h per day compared with women younger than 50 years and working for <9 h, whereas this was not significant among men [39]. These previous study findings support our findings.

It is difficult to explain the association between hours of work and obesity unidimensionally. Extended hours of work are associated with a variety of behaviors including dietary habits and activity patterns (such as lack of physical activity, increase in the intake of fast foods, and lack of sleep), and if such dietary and behavioral changes persist, the metabolism is negatively affected (such as obesity) [20,40]. Hyun et al. found that while 63.4% of workers with obesity tried to control their weight, the OR of weight control effort was 2.4-times higher among subjects who worked for 40–49 h per week than among those who worked for >60 h per week [41]. It has also been reported that the need for a healthcare utilization is not satisfied among individuals who work for extended hours [42]. These findings show that extended hours of work make it difficult to engage in efforts in order to control weight and utilize healthcare services. In a study which analyzed the data from the Korean Working Conditions Survey, a reduction in working hours improved the indices of a work–life balance and a satisfaction with working conditions [43].

According to the “2018 Work, Family Balance Indicator” reported by Statistics Korea [44], the employment rate among women was 50.8%, showing an increasing trend. The mean weekly number of working hours was 45.2 among men and 39.6 among women. However, as many as 24.9% of the subjects in our study worked for ≥50 h per week. In Korea, the gender difference in housework responsibility is quite large. Although more than 50% believe in a fair division of housework, it is reported that only 20% share domestic responsibilities fairly. Thus, working women in Korea may not get enough rest because of housework and child rearing that they are responsible for after returning home from work. In consideration of such cultural features, the society as a whole should take an interest in weight management among working women.

The present study showed that the work pattern was associated with obesity among working women. The OR of night or shift workers obesity was 1.21, compared with day or evening workers. In a systematic review of 26 studies on the relationship between shift work and weight gain, the risk ratio (RR) for overweight was 1.25 and the RR for obesity was 1.17 in shift work, indicating a positive relationship between shift work and weight gain [12]. Another systematic review of 28 studies on the association between shift work and obesity found that the likelihood of overweight and obesity increased by 1.23 times among night workers compared with day workers [45]. These results are in line with our study finding. Approximately 30% of Korean female workers are pink-collar workers, employed in the service or sales sector [46]. Pink-collar workers are more likely to work in shifts. Working at night or in shift work changes the circadian rhythm, increasing fatigue and stress. Eating or sleeping during night shift work can interfere with the energy metabolism by weakening the digestive system [47]. Furthermore, shift work can increase physiological problems among women like irregular menstruation [48], compounding the symptoms of a metabolic syndrome, such as obesity.

In addition, among women, factors related to changes in sex hormones (for example, menopause) can impact the obesity [28,49]. In our study, such a variable was considered important which is why the menopausal status was included in the analysis. The association between weight category and menopause was found to be significant. In the logistic regression analysis, the OR of obesity among post-menopausal women was 1.06 (95% CI, 0.88–1.29) compared to pre-menopausal women, but the association was no longer significant. To our knowledge, this variable has rarely been controlled in studies on obesity among women. Rather, most studies merely mentioned the failure to control the variable as a limitation. In light of this, our study finding has important implications.

A major strength of our study is that the association between occupational characteristics and overweight/obesity was investigated using a nationally representative, large-scale sample. Another strength is that a variable related to the sex hormone level, which may be an influential factor in obesity among women, was included, and the results were derived while controlling the variable in the analysis. A notable limitation of the study is that the findings may not be generalized to other populations or the general public due to the study population consisting of Korean female workers. Additionally, the study of male obesity is valuable, but we only studied women. There is a lack of research on the relationship between occupational factors and obesity in Korean women. In particular, Korean women are more likely than men to work both at the workplace and at home, since women still take much responsibility for housework, so these factors can lead to changes in life and contribute to obesity. In addition, there are cases where men and women need to apply differently, such as hormone effects, in the use of correction variables. Another limitation is that while the BMI was computed using measurements of height and weight among the subjects, thereby increasing the accuracy of the variable, other variables were based on self-reports and thus their accuracy may have not been high. Lastly, a causal relationship between the number of working hours and obesity was not assessed in this study. In the future, longitudinal studies should be conducted to assess the causality between the variables.

## 5. Conclusions

Our study demonstrated that occupational characteristics are linked to overweight and obesity even after controlling for demographic, behavioral, and health-related characteristics. Specifically, the number of working hours, work pattern, and job category were each found to be associated with being overweight or obese, after controlling for confounding variables that may also involve factors of overweight and obesity among working women. On the basis of the findings, it is suggested that occupational characteristics, including the number of working hours, work pattern, and job category, should be considered in the prevention and management of obesity among working women. Further research should be conducted on these influential factors.

## Figures and Tables

**Table 1:** Weight category and demographic, behavioral, health-related, and occupational characteristics.

Variables	Weight Category				
	Total (*n* = 2090)	Underweight (*n* = 143)	Normal weight (*n* = 1062)	Overweight (*n* = 421)	Obesity (*n* = 464)
**Age, yrs** *	38.8 ± 0.197				
19–29	434(26.3)	62(14.2)	253(58.4)	52(12.1)	67(15.3)
30–39	557(24.8)	53(9.1)	318(54.9)	94(17.7)	92(18.4)
40–49	610(26.9)	21(3.2)	297(48.7)	142(23.3)	150(24.8)
≥50	489(19.3)	7(1.8)	194(38.5)	133(27.6)	155(32.0)
**Education**					
≤Elementary	171(7.4)	2(1.8)	55(30.4)	43(24.7)	71(43.1)
Middle school	172(8.1)	2(1.5)	68(39.7)	43(23.8)	59(35.0)
High school	772(37.6)	43(6.2)	352(45.8)	173(22.2)	204(25.9)
≥College	975(46.8)	96(10.1)	587(60.0)	162(16.2)	130(13.5)
**Income**					
First quartile	109(5.4)	4(5.6)	48(44.0)	20(17.4)	37(33.1)
Second quartile	481(23.4)	37(8.5)	210(43.8)	91(18.7)	143(29.0)
Third quartile	705(34.4)	42(6.3)	360(52.2)	138(18.6)	165(22.9)
Fourth quartile	795(36.9)	60(7.7)	444(54.9)	172(21.9)	119(15.4)
**Marital status**					
Married	1576(71.0)	71(4.3)	765(48.1)	353(22.3)	387(25.2)
Single	514(29.0)	72(14.5)	297(57.4)	68(13.6)	77(14.5)
**Smoking**					
Yes	115(6.6)	10(9.3)	47(39.3)	22(18.0)	36(33.4)
No	1975(93.4)	133(7.1)	1015(51.6)	399(19.9)	428(21.3)
**Alcohol use**					
Yes	1087(53.0)	62(6.0)	573(52.3)	215(20.5)	237(21.2)
No	1003(47.0)	80(8.7)	490(49.2)	206(19.0)	227(23.2)
**Physical activity**					
Yes	741(37.6)	38(5.8)	366(48.4)	165(22.7)	172(23.1)
No	1349(62.4)	105(8.2)	696(52.2)	256(18.1)	292(21.5)
Sleep duration, h *	6.8 ± 0.015				
<7	874(41.3)	52(5.9)	415(48.4)	199(22.4)	208(23.3)
≥7	1216(58.7)	91(8.3)	647(52.5)	222(18.0)	256(21.3)
Daily energy intake, kcal *	1799.6 ± 9.132				
<2000	1439(68.2)	95(7.1)	720(50.3)	291(20.0)	333(22.6)
2000–2499	360(18.1)	23(7.4)	189(52.0)	75(20.8)	73(19.8)
≥2500	291(13.7)	25(8.1)	153(51.8)	55(17.5)	58(22.6)
**Stress level**					
High	648(32.4)	48(7.0)	332(51.3)	126(19.5)	142(22.2)
Low	1442(67.6)	95(7.4)	730(50.6)	295(19.9)	322(22.1)
**Hypertension**					
Yes	241(10.8)	1(0.1)	81(33.8)	39(16.4)	120(49.7)
No	1849(89.2)	142(8.2)	981(52.9)	382(20.2)	344(18.8)
**Diabetes**					
Yes	59(2.7)	2(2.1)	11(19.5)	15(27.0)	31(51.4)
No	2031(97.3)	141(7.4)	1051(51.7)	406(19.6)	433(21.3)
**Dyslipidemia**					
Yes	400(17.6)	8(1.7)	125(32.0)	92(22.4)	175(43.9)
No	1690(82.4)	135(8.5)	937(54.8)	329(19.3)	289(17.4)
**Menopause**					
Yes	461(18.8)	7(1.9)	187(39.3)	123(26.1)	144(32.7)
No	1629(81.2)	136(8.5)	875(53.5)	298(18.3)	320(19.7)
Working hours/week *	45.4 ± 0.097				
<40	322(16.0)	23(7.6)	169(53.9)	70(22.1)	60(16.3)
40–49	1247(59.1)	86(7.2)	658(52.0)	239(19.0)	264(21.8)
50–59	334(16.1)	27(9.7)	161(50.2)	66(18.5)	80(21.7)
≥60	187(8.8)	7(2.9)	74(38.4)	46(23.5)	60(35.3)
**Work pattern**					
Night or shift worker	125(6.4)	9(9.3)	62(46.2)	22(16.8)	32(27.9)
Day or evening worker	1965(93.6)	134(7.1)	1000(51.1)	399(20.0)	432(21.7)
**Working status**					
Permanent	1677(80.2)	114(7.0)	881(52.6)	330(19.4)	352(21.1)
Temporary	312(15.4)	25(10.1)	140(44.2)	69(22.2)	78(23.5)
Daily	101(4.4)	4(3.4)	41(42.2)	22(19.2)	334(35.2)
**Job category**					
Blue collar	504(23.7)	18(4.3)	208(40.0)	122(23.9)	156(31.8)
Pink collar	431(20.9)	22(5.7)	190(43.4)	98(23.8)	121(27.2)
White collar	1155(55.4)	103(9.2)	664(58.2)	201(16.6)	187(16.0)

* Values are presented as the mean ± standard error; BMI, body mass index; underweight: BMI <18.5 kg/m^2^; normal weight: BMI 18.5–22.9 kg/m^2^; overweight: BMI 23.0–24.9 kg/m^2^; obesity: BMI ≥25 kg/m^2^.

**Table 2:** Associations between weight category and demographic, behavioral, health-related, and occupational characteristics.

Variables	Weight Category				
	Underweight and Normal Weight (*n* = 1205)	Overweight (*n* = 421)	Obesity (*n* = 464)	χ^2^	*p*-Value
**Age, years**					
19–29	315(72.6)	52(12.1)	67(15.3)	117.720	<0.001 ^a,b^
30–39	371(63.9)	94(17.7)	92(18.4)		
40–49	318(51.8)	142(23.3)	150(24.8)		
≥50	201(40.3)	133(27.6)	155(32.0)		
**Education**					
≤Elementary	57(32.2)	43(24.7)	71(43.1)	148.552	<0.001 ^a,b,c^
Middle school	70(41.2)	43(23.8)	59(35.0)		
High school	395(51.9)	173(22.2)	204(25.9)		
≥College	683(70.1)	162(16.4)	130(13.5)		
**Income**					
First quartile	52(29.5)	20(17.4)	37(33.1)	42.116	<0.001 ^b,c^
Second quartile	247(52.3)	91(18.7)	143(29.0)		
Third quartile	402(58.5)	138(18.6)	165(22.9)		
Fourth quartile	504(62.6)	172(21.9)	119(15.4)		
**Marital status**					
Married	836(52.4)	353(22.3)	387(25.2)	67.000	<0.001 ^a,b^
Single	369(71.9)	68(13.6)	77(14.5)		
**Smoking**					
Yes	57(48.6)	22(18.0)	36(33.4)	10.999	<0.001 ^b,c^
No	1148(58.8)	399(19.9)	428(21.3)		
**Alcohol use**					
Yes	635(58.3)	215(20.5)	237(21.2)	1.625	0.155
No	570(57.9)	206(19.0)	227(23.2)		
**Physical activity**					
Yes	404(54.2)	165(22.7)	172(23.1)	9.251	<0.001 ^a^
No	801(60.4)	256(18.1)	292(21.5)		
**Sleep duration, hours**					
<7	467(54.3)	199(22.4)	208(23.3)	9.680	<0.001 ^a,b^
≥7	738(60.8)	222(18.0)	256(21.3)		
**Daily energy intake (kcal)**					
<2000	815(57.4)	291(20.0)	333(22.6)	2.456	0.008^c^
2000–2499	212(59.3)	75(20.8)	73(19.8)		
≥2500	178(59.9)	55(17.5)	58(22.6)		
**Stress level**					
High	380(58.3)	126(19.5)	142(22.2)	0.055	0.782
Low	825(58.0)	295(19.9)	322(22.1)		
**Hypertension**					
Yes	82(33.9)	39(16.4)	120(49.7)	114.067	<0.001 ^a,b,c^
No	1123(61.0)	382(20.2)	344(18.8)		
**Diabetes**					
Yes	13(21.6)	15(27.0)	31(51.4)	36.955	<0.001 ^a,b,c^
No	1192(59.1)	406(19.6)	433(21.3)		
**Dyslipidemia**					
Yes	133(33.7)	92(22.4)	175(43.9)	143.083	<0.001 ^a,b,c^
No	1072(63.3)	329(19.3)	289(17.4)		
**Menopause**					
Yes	194(41.2)	123(26.1)	144(32.7)	58.124	<0.001 ^a,b^
No	1011(62.0)	298(18.3)	320(19.7)		
**Working hours/week**					
<40	192(61.6)	70(22.1)	60(16.3)	32.307	<0.001 ^a,b,c^
40–49	744(59.2)	239(19.0)	264(21.8)		
50–59	188(59.8)	66(18.5)	80(21.7)		
≥60	81(41.3)	46(23.5)	60(35.3)		
**Work pattern**					
Night or shift worker	71(55.3)	22(16.8)	32(27.9)	3.008	0.003 ^b,c^
Day or evening worker	1134(58.3)	399(20.0)	432(21.7)		
**Working status**					
Permanent	995(59.5)	330(19.4)	352(21.1)	13.283	<0.001 ^a,b,c^
Temporary	165(54.2)	69(22.2)	78(23.5)		
Daily	45(45.6)	22(19.2)	34(35.2)		
**Job category**					
Blue collar	226(44.3)	122(23.9)	156(31.8)	98.970	<0.001 ^a,b,c^
Pink collar	212(49.0)	98(23.8)	121(27.2)		
White collar	767(67.4)	201(16.6)	187(16.0)		

The Bonferroni correction set the significance cut-off at 0.05/3. ^a^ Bonferroni correction between underweight and normal weight groups and overweight groups. ^b^ Bonferroni correction between underweight and normal weight groups and obesity groups. ^c^ Bonferroni correction between overweight groups and obesity groups.

**Table 3:** Results of multiple logistic regression analysis of the association between occupational characteristics and overweight/obesity.

Variables	Overweight		Obesity	
	OR	95% CI	OR	95% CI
**Age, years** (ref. 19–29)				
30–39	1.54	1.35–1.76	1.05	0.85–1.28
40–49	1.96	1.60–2.41	0.96	0.77–1.20
≥50	2.73	2.26–3.29	0.83	0.66–1.03
**Education** (ref. college)				
≤Elementary	1.57	1.17–2.11	2.58	1.84–3.63
Middle school	1.16	0.99–1.36	2.03	1.39–2.97
High school	1.27	1.11–1.45	1.71	1.34–2.18
**Income** (ref. fourth quartile)				
First quartile	0.62	0.48–0.79	1.46	1.18–1.81
Second quartile	0.85	0.71–1.03	1.61	1.28–2.04
Third quartile	0.81	0.71–0.93	1.32	1.17–1.50
**Marital status** (ref. single)				
Married	0.99	0.90–1.09	1.25	1.01–1.54
**Smoking** (ref. no)				
Yes	1.12	0.94–1.33	1.59	1.29–1.94
**Physical activity** (ref. no)				
Yes	1.44	1.36–1.52	1.28	1.06–1.53
**Sleep duration, hours** (ref. **≥7**)				
<7	1.23	1.14–1.34	1.02	0.92–1.13
**Daily energy intake, kcal** (ref. <2000)				
2000–2499	0.91	0.77–1.07	0.90	0.81–1.01
≥2500	1.02	0.90–1.17	1.14	0.98–1.34
**Hypertension** (ref. no)				
Yes	0.70	0.55–0.88	2.08	1.74–2.48
**Diabetes** (ref. no)				
Yes	2.49	1.80–3.44	2.57	1.79–3.69
**Dyslipidemia** (ref. no)				
Yes	1.57	1.37–1.78	3.16	2.70–3.68
**Menopause** (ref. no)				
Yes	0.84	0.69–1.03	1.06	0.88–1.29
**Working hours/week** (ref. <40)				
40–49	1.03	0.87–1.22	1.56	1.17–2.08
50–59	0.93	0.80–1.08	1.38	1.03–1.85
≥60	1.38	1.06–1.81	2.68	2.13–3.36
**Work pattern** (ref. day or evening worker)				
Night or shift worker	0.79	0.62–1.02	1.21	1.01–1.45
**Working status** (ref. permanent)				
Temporary	1.16	1.01–1.34	0.97	0.78–1.19
Day-to-day	0.77	0.55–1.06	1.03	0.76–1.40
**Job category** (ref. white collar)				
Blue collar	1.35	1.16–1.57	1.13	0.92–1.38
Pink collar	1.41	1.15–1.72	1.07	0.73–1.56

The reference group was normal weight (BMI, 18.5–22.9 kg/m^2^); adjusted ORs from multivariate logistic regression analysis. Adjusted for age, education, income, marital status, smoking, physical activity, daily energy intake, sleep duration, hypertension, diabetes, dyslipidemia, and menopause.
